# Pulmonary hemorrhage after pulmonary endarterectomy in a patient with chronic thromboembolic pulmonary hypertension: a case report

**DOI:** 10.1051/ject/2025017

**Published:** 2025-12-17

**Authors:** Gleb Moroz, Alexander Edemskiy, Igor Kornilov, Ramzil Nasyrtdinov, Alexander Chernyavskiy

**Affiliations:** 1 E. Meshalkin National Medical Research Center 15 Rechkunovskaya Street Novosibirsk 630055 Russia; 2 Department of Anesthesiology and Perioperative Medicine, Milton S. Hershey Medical Center, Penn State University 500 University Dr Hershey 17033 USA; 3 Novosibirsk State Medical University 52 Krasny Prospekt Novosibirsk 630091 Russia

**Keywords:** Chronic thromboembolic pulmonary hypertension, Pulmonary endarterectomy, Pulmonary hemorrhage, Hemoptysis, ECMO, Cardiac surgery

## Abstract

Massive pulmonary hemorrhage is the severe complication of pulmonary endarterectomy, associated with high mortality rates. ECMO may be a life-saving option for patients with pulmonary hemorrhage. In this case, we have described the successful sequential application of both VA and VV ECMO modalities. VA ECMO was employed initially to reduce blood flow to the right heart and pulmonary artery. Once hemodynamic stability was achieved, VV-ECMO was utilized to support severe respiratory failure. A six-month follow-up demonstrated good clinical outcome, with no evidence of pulmonary artery rethrombosis.

## Introduction

Chronic thromboembolic pulmonary hypertension (CTEPH) is a rare, severe complication of acute pulmonary embolism. Unresolved thrombi become organized and fibrotic and may lead to chronic pulmonary hypertension, right heart failure, and death [1]. Pulmonary endarterectomy (PEA) is the gold standard of treatment for CTEPH and leads to a reduction of pulmonary hypertension and right heart failure (class of recommendation and level of evidence IB) [1–3]. A pulmonary hemorrhage is a severe complication of PEA and a significant treatment challenge. Here, we present a case of severe endobronchial hemorrhage following PEA.

## Materials and methods

**Figure 1:**
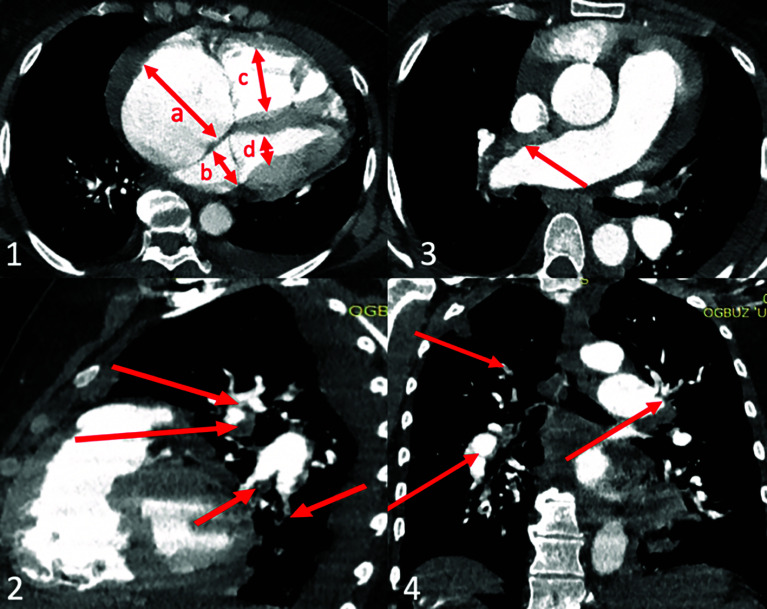
CT-angiogram: 1 – axial view shows right atrium (a) and right ventricle (c) enlargement compare to left atrium (b) and left ventricle (d); 2 – sagittal view shows organized thrombi in lobar and segmental branches of left pulmonary artery (red arrows); 3 – axial view shows proximal organized thrombus in right main pulmonary artery (red arrow); 4 – coronal view shows organized thrombi in right and left lobar pulmonary artery branches (red arrows).

Written informed consent for publication was obtained from a patient. Patient B., a 51-year-old woman with a known history of CTEPH and thrombophilia due to protein C deficiency, was referred to our tertiary cardiac surgery center for further management. On admission, the pulse oximetry was 94% on room air. Echocardiography showed significant right ventricle (RV) systolic dysfunction with fractional area change (FAC) of 22%, end-diastolic right ventricle volume of 106 mL, tricuspid annular plane systolic excursion (TAPSE) of 1.1 cm, with moderate tricuspid regurgitation. Computer tomography (CT) pulmonary angiography confirmed a proximal form of CTEPH (Figure 1). Right heart catheterization showed a pulmonary artery pressure (PAP) 97/45/62 mmHg and the pulmonary vascular resistance (PVR) of 888 dynes/s/cm^−5^.

**Figure 2:**
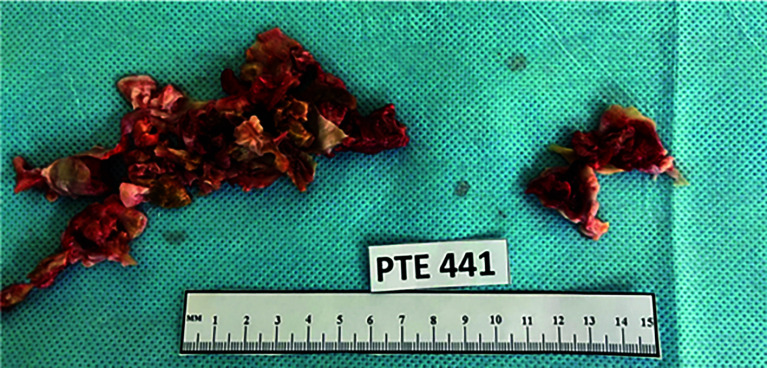
Thrombi from pulmonary arteries.

The PEA was performed using a standard technique under cardiopulmonary bypass (CPB) with deep hypothermic circulatory arrest (DHCA) at 18 °C. The removed chronic organized thrombi are shown in Figure 2.

The DHCA time was 42 min (20 min for the left PA and 22 min for the right PA). After releasing the tourniquets from the superior and inferior vena cavae and restoring blood flow to the right heart, a massive hemorrhage through the endotracheal tube was noted. The attempt to wean from CPB was unsuccessful due to worsening pulmonary hemorrhage with severe refractory arterial hypotension and desaturation. A decision was made to switch to the veno-arterial (VA) extracorporeal membrane oxygenation (ECMO).

**Figure 3:**
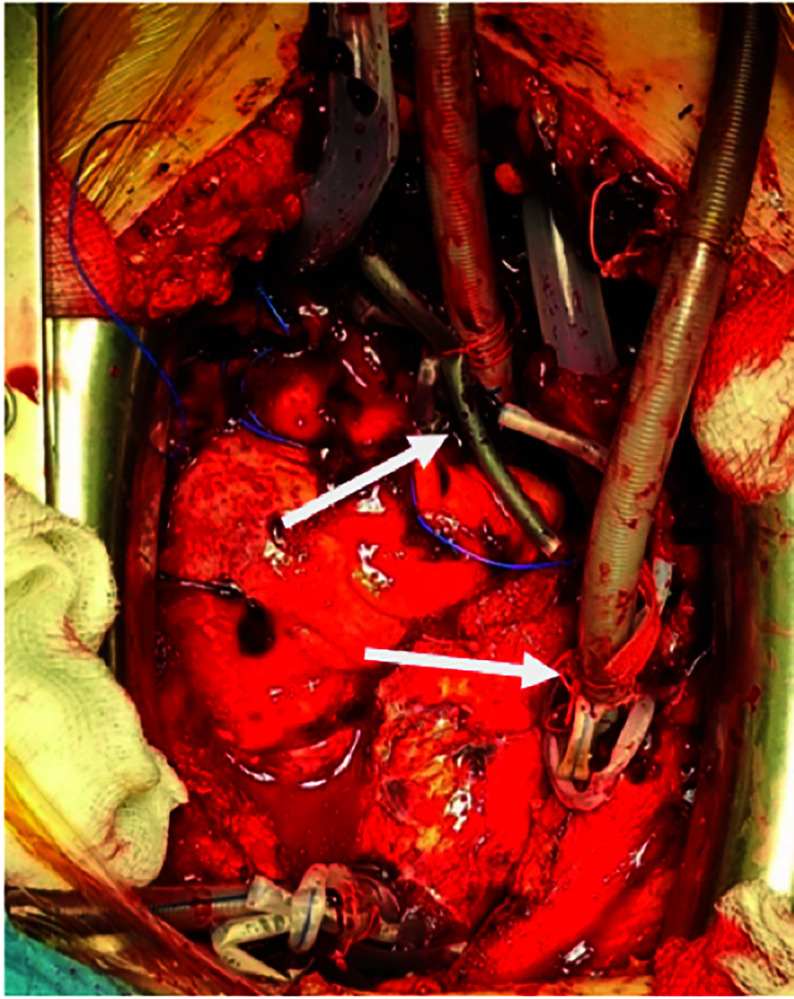
Central VA ECMO cannulation with the tourniquets on superior and inferior vena cava (arrows).

**Figure 4:**
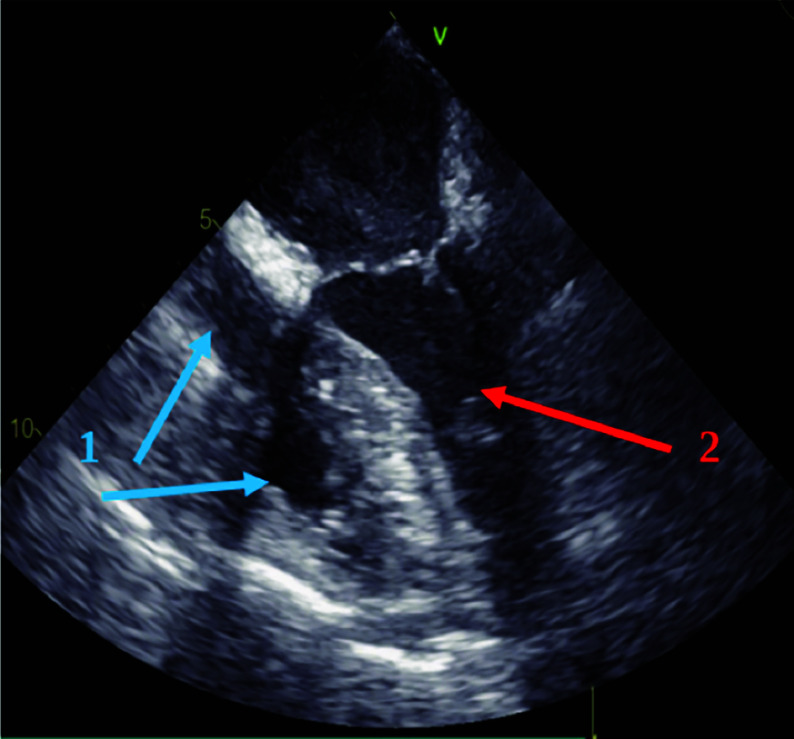
Transesophageal echocardiography picture. The right chambers of heart are collapsed due to snared superior and inferior cava veins (1, blue arrows); reduced end diastolic volume of left ventricle (2, red arrow).

CPB cannulas were connected to the VA ECMO circuit, and the superior and inferior vena cavae were snared to minimize a blood flow to the right heart and the pulmonary artery (Figure 3). In this ECMO configuration, end-diastolic volumes of the right ventricle (RV) and left ventricle (LV) were 16 mL and 38 mL, respectively (Figure 4).

**Figure 5:**
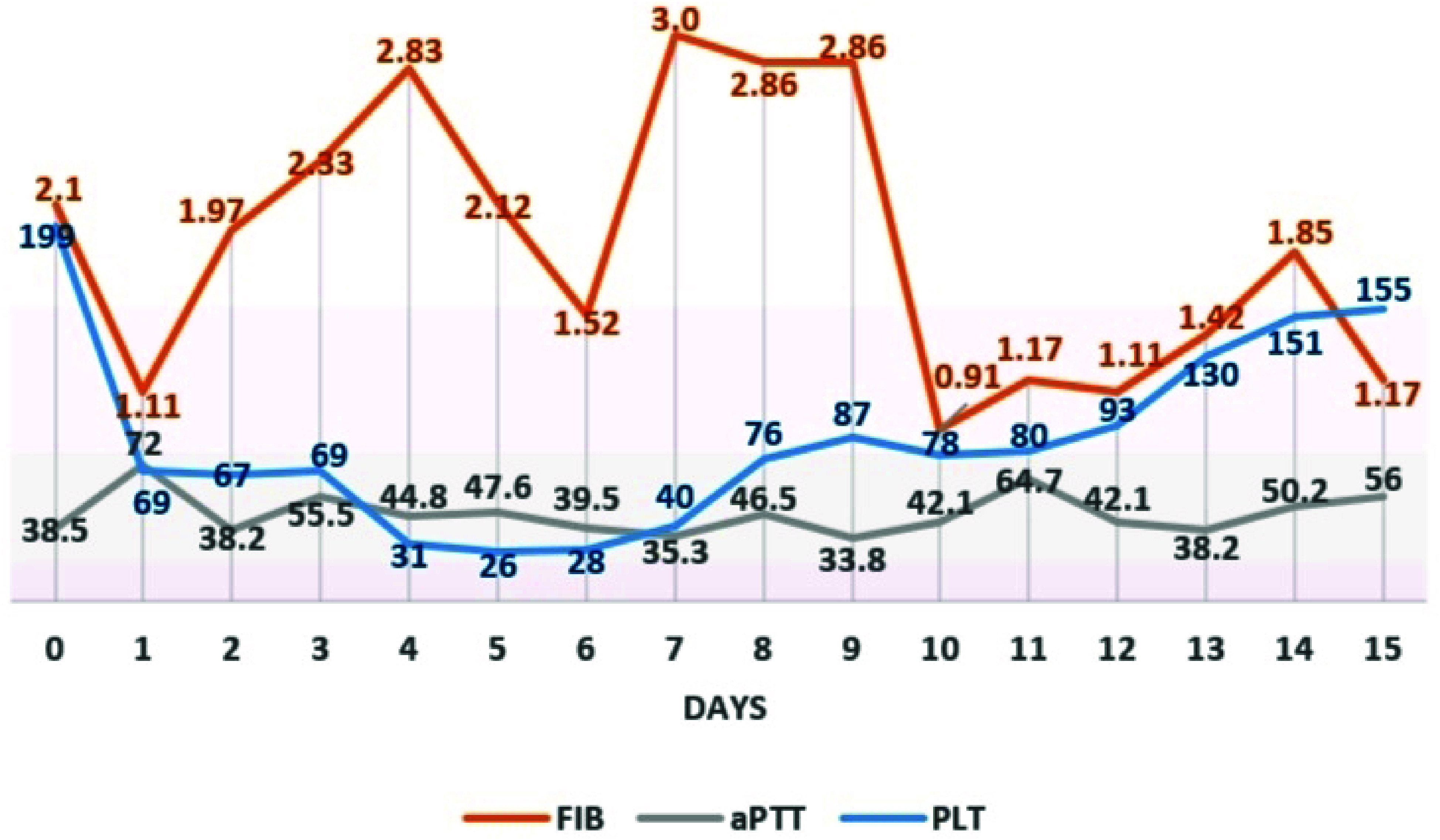
Trends in coagulation parameters during the perioperative period. Day 0 corresponds to the preoperative baseline; days 1 to 14 represent the period of ECMO support; and day 15 reflects values following ECMO wean. PLT, platelet count (×10^9^/L); FIB, fibrinogen concentration (g/L); aPTT, activated partial thromboplastin time (seconds).

Lung ventilation was discontinued since ECMO had started. Heparin was neutralized by protamine. Intraoperative blood loss through the endotracheal tube was approximately 1000 mL. To correct anemia, two units of red blood cells (RBC) were transfused in the operating room. The patient was transferred to the intensive care unit (ICU). A bronchoscopy revealed clots and blood in both the right and left main bronchi, with the lobar bronchi completely obstructed by clots. Multiple bronchoscopies were performed to remove the clots and wash out the blood from the bronchi. The patient remained off mechanical ventilation for 2 days, and, as no signs of active bleeding were observed after this period, the protective lung ventilation was resumed. During the postoperative period, the patient received four units of RBCs, eight units of fresh frozen plasma (FFP), and one unit of platelets. Heparin infusion was initiated on the third day, targeting an activated clotting time (ACT) of 180–200 s, but was later switched to bivalirudin infusion due to thrombocytopenia. Changes in fibrinogen, platelet count, and activated partial thromboplastin time (APTT) during the postoperative period are presented in Figure 5.

The attempt to wean from ECMO on the postoperative day four was unsuccessful due to severe desaturation (SatO_2_ = 83%) and hypoxemia (PaO_2_/FiO_2_ = 66 mm Hg with FiO_2_ = 100%). The patient’s hemodynamics were stable: blood pressure was 106/68 mmHg (mean 79), pulmonary artery pressure was 46/28 mmHg (mean 25 mmHg), cardiac output was 5.2 L/min, PVR was 384 dynes/s/cm^−5^, left ventricle ejection fraction (LVEF) was 68%, and RV FAC was 33%. The patient was switched to the peripheral veno-venous (VV) ECMO (femoral-jugular) and the chest was closed. Physiotherapy and mobilization were started the following day. A tracheostomy was performed on the seventh postoperative day. The VV ECMO was discontinued on postoperative day 14 – the patient was ventilated with FiO_2_ = 50% and had SatO_2_ = 99.5%, PaO_2_ = 117 mmHg, and PaCO_2_ = 33.7 mmHg. The patient was successfully weaned from mechanical ventilation 6 days later. The patient was transferred to the ward unit with good saturation without O_2_ therapy on the postoperative day 25 and was discharged from the hospital in good condition. A direct oral anticoagulant was chosen for long-term anticoagulation due to her protein C deficiency.

**Figure 6:**
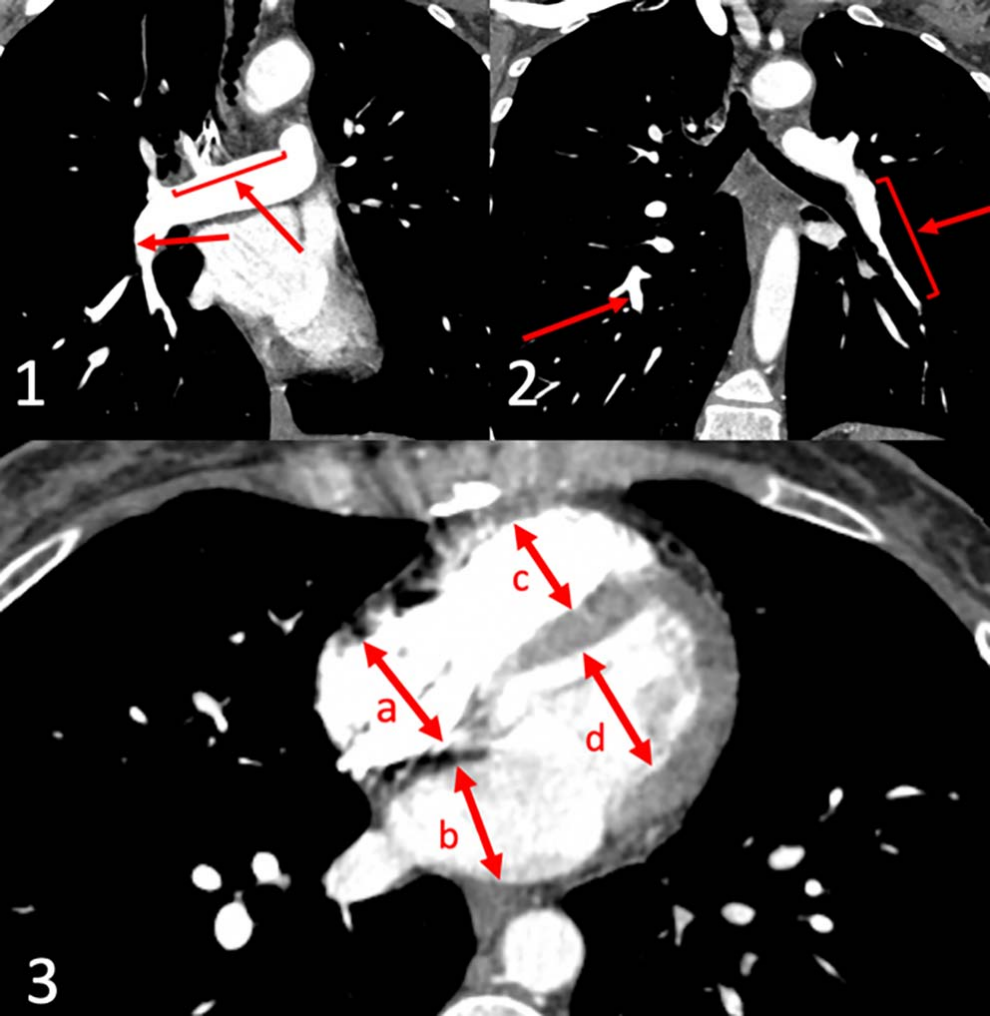
CT angiogram 6 months after discharge: 1 – coronal view shows absence of thrombi after pulmonary endarterectomy in right main and left inferior lobar pulmonary artery (red arrows); 2 – coronal view shows absence of thrombi after pulmonary endarterectomy in left inferior and right inferior lobar pulmonary arteries (red arrows); 3 – axial view shows right atrium (a) and right ventricle (c) remodeling after surgery compare to left atrium (b) and left ventricle (d).

A follow-up examination at 6 months demonstrated a good clinical outcome. The right heart catheterization revealed PAP of 41/17/25 mmHg, the pulmonary artery wedge pressure of 10 mmHg, cardiac output of 5.8 L/min, and the PVR of 206 dynes/s/cm^−5^. CT pulmonary angiography showed no evidence of pulmonary artery rethrombosis, and right heart remodeling was also observed (Figure 6).

## Discussion

Massive pulmonary hemorrhage is one of the most severe complications of surgery for chronic thromboembolic pulmonary hypertension, with an incidence ranging from 0.5% to 2% [4]. Currently, there are no prognostic criteria for the development of this complication following PEA.

To date, several case reports have been published with varying treatment approaches for the pulmonary hemorrhages following PEA [4–6]. Possible treatment options include mechanical ventilation with high positive end-expiratory pressure, endoscopic bronchial obstruction, and procoagulation therapy. The cases of successful application of Fogarty catheter [7] or using local hemostatic material (Surgicel) or biological glue (BioGlue) are also described [5, 7]. Some authors used vasoconstrictors, such as vasopressin and epinephrine, administered through the endotracheal tube, along with intensive procoagulation therapy (FFP, platelets) [8]. According to our experience, these methods help to localize the origin of the moderate bleeding, but are less effective for massive endobronchial hemorrhage when both lungs are filled with blood.

Nowadays, ECMO is considered the primary intervention for this life-threatening complication. Both VV ECMO and VA ECMO have been described for use in these scenarios. According to data from Thistlethwaite et al., out of 20 cases of pulmonary hemorrhage treated with ECMO, six patients survived [9]. Berman et al. have shown the data where four out of seven patients with the pulmonary hemorrhage survived [10]. The use of VV ECMO in patients with pulmonary hemorrhage, according to Yıldızeli et al., can decrease the hospital mortality rate by 50% [6]. Kabadi et al. published outcomes in 58 patients with various severities of pulmonary hemorrhage after PEA with a mortality rate of 13.8% and identified age, female sex, history of preoperative hemoptysis, and higher preoperative PVR as risk factors for the pulmonary hemorrhage [11]. The extensive experience is presented from the Royal Papworth Hospital, where 31 patients were treated with ECMO, with a survival rate of 51.8% [12]. The authors in most cases used ECMO as a short-term concept to reverse heparin and normalize hemostasis following CPB, and the weaning from ECMO typically occurred in the operating theatre [12, 13].

In our case of massive endobronchial hemorrhage, in which both lungs were immediately filled with blood, VA ECMO was utilized not only to replace lung and heart function but also to reduce blood flow to the right heart and pulmonary artery. This was achieved by snaring the inferior and superior vena cava. Additionally, the application of ECMO allowed for heparin neutralization and initiation of procoagulant therapy with FFP and platelets. Once the pulmonary hemorrhage was controlled, a conversion to peripheral VV-ECMO was performed. This approach facilitated the replacement of respiratory function until lung recovery, chest closure to minimize the risk of surgical site infection and bleeding, and early mobilization.

There was a risk of recurrent thrombosis in the pulmonary arteries, especially in the endarterectomy areas, due to reduced pulmonary blood flow during the VA ECMO and the use of procoagulation therapy. However, the CT pulmonary angiography at the 6-month follow-up showed no evidence of rethrombosis.

In conclusion, ECMO is a treatment option for the management of severe pulmonary hemorrhage following PEA. Cannulation of both vena cavae may reduce the pulmonary blood flow and provide an effective means to control the severe pulmonary hemorrhage.

## Data Availability

All available data are incorporated into the article.

## References

[1] Humbert M, Kovacs G, Hoeper M, et al. ESC/ERS Guidelines for the diagnosis and treatment of pulmonary hypertension: Developed by the task force for the diagnosis and treatment of pulmonary hypertension of the European Society of Cardiology (ESC) and the European Respiratory Society (ERS). Endorsed by the International Society for Heart and Lung Transplantation (ISHLT) and the European Reference Network on rare respiratory diseases (ERN-LUNG). Eur Heart J. 2022;2022:3618–3731.

[2] Delcroix M, Torbicki A, Gopalan D, et al. ERS statement on chronic thromboembolic pulmonary hypertension. Eur Respir J. 2021;57:2002828. 33334946 10.1183/13993003.02828-2020

[3] Madani MM, Wiedenroth CB, Jenkins DP, et al. Pulmonary thromboendarterectomy: the potentially curative treatment of choice for chronic thromboembolic pulmonary hypertension. Ann Thorac Surg. 2025;119(4):756–767. 39265713 10.1016/j.athoracsur.2024.07.052

[4] Jenkins D, Martinez G, Salaunkey K, et al. Perioperative management in pulmonary endarterectomy. Semin Respir Crit Care Med. 2023;44:851–865. 37487525 10.1055/s-0043-1770123

[5] Kanchi M, Nair HC, Natarajan P, et al. Management of intrapulmonary hemorrhage in patients undergoing pulmonary thrombo-endarterectomy. Ann of Cardiac Anaesth. 2021;24:384–388. 10.4103/aca.ACA_191_20 PMC8404590 34269276

[6] Yıldızeli SO, Erkılınç A, Yanartas M, et al. Perioperative management of massive pulmonary hemorrhage after pulmonary endarterectomy., Turk Gogus Kalp Damar Cerrahisi Derg. 2018;26:429–435. 32082774 10.5606/tgkdc.dergisi.2018.15404 PMC7018270

[7] Morsolini M, Azzaretti A, Orlandoni G, et al. Airway bleeding during pulmonary endarterectomy: the “bubbles” technique. J Thorac Cardiovasc Surg Surgery. 2013;145(5):1409–1410. 10.1016/j.jtcvs.2012.12.045 23312345

[8] Manecke Jr G, Kotzur A, Atkins G, et al. Massive pulmonary hemorrhage after pulmonary thromboendarterectomy: case reports. Anesth Analg. 2004;99:672–675. 15333391 10.1213/01.ANE.0000130000.15786.82

[9] Thistlethwaite PA, Madani MM, Kemp MM, et al. Venovenous extracorporeal life support after pulmonary endarterectomy: indications, techniques, and outcomes. Ann Thorac Surg. 2006; 82:2139–2145. 17126125 10.1016/j.athoracsur.2006.07.020

[10] Berman M, Tsui S, Vuylsteke A, et al. Successful extracorporeal membrane oxygenation support after pulmonary thromboendarterectomy. Ann Thorac Surg. 2008;86:1261–1267. 18805172 10.1016/j.athoracsur.2008.06.037

[11] Kabadi AA, Fernandes TM, Papamatheakis DG, et al. Airway hemorrhage complicating pulmonary thromboendarterectomy: risk factors and outcomes. Ann Thorac Surg. 2023;116(1):121–128. 36368350 10.1016/j.athoracsur.2022.11.003

[12] Chia AXF, Valchanov K, Ng C, et al. Perioperative extracorporeal membrane oxygenation support for pulmonary endarterectomy: a 17-year experience from the UK national cohort. J Heart Lung Transplant. 2024;43:241–250. 37730188 10.1016/j.healun.2023.09.008

[13] Guth S, Wiedenroth CB, Wollenschläger M, et al. Short-term venoarterial extracorporeal membrane oxygenation for massive endobronchial hemorrhage after pulmonary endarterectomy. J Thorac Cardiovasc Surg. 2018;155:643–649. 29033041 10.1016/j.jtcvs.2017.09.045

